# Syntaxin-7 promotes EMT and tumor progression via NF-κB signaling and is associated with macrophage infiltration: pan-cancer analysis and experimental validation in hepatocellular carcinoma

**DOI:** 10.1186/s12885-025-14819-0

**Published:** 2025-09-25

**Authors:** Langhuan Lei, Wei Shi, Xing Yang, Jiali Lin, Qiuyu Liang, Xiaozhi Huang, Liuxian Pan, Wei Li

**Affiliations:** 1 Research Center of Health Management, Guangxi Zhuang Autonomous Region People’s Hospital and Guangxi Academy of Medical Sciences, Nanning, 530012 China; 2 Department of Health Management, Guangxi Zhuang Autonomous Region People’s Hospital and Guangxi Academy of Medical Sciences, Nanning, 530012 China

**Keywords:** STX7, Pan-cancer, Immune infiltration, EMT, Hepatocellular carcinoma

## Abstract

**Background:**

Syntaxin-7 (STX7), a membrane trafficking-related gene, has been implicated in various cancers, but its specific role in hepatocellular carcinoma remains unclear. There has been no pan-cancer analysis examining the immunological function and prognostic significance of STX7 so far.

**Methods:**

We chose STX7 for an in-depth investigation to explore its expression patterns, prognostic significance, enriched pathways, and immune infiltration across various cancers. The transcriptional landscape of STX7 was examined at both single-cell and spatial levels, and a combination of in vitro and in vivo experiments was conducted to further validate its functional roles.

**Results:**

STX7 was significantly upregulated in a wide range of cancer types and was associated with poor prognosis. Additionally, STX7 was correlated with immune cell infiltration and key immune regulators. Enrichment analysis further underscored the potential role of STX7 in immune evasion and tumor progression. Single-cell and spatial transcriptome analyses revealed its specific expression in macrophages. Functional experiments demonstrated that STX7 knockout suppressed hepatocellular carcinoma proliferation and migration, while inhibiting epithelial-mesenchymal transition (EMT) via NF-κB signaling.

**Conclusion:**

STX7 promotes EMT and tumor progression via NF-κB signaling, with a strong association to macrophage infiltration in cancers, including hepatocellular carcinoma, highlighting its potential as a prognostic biomarker and therapeutic target.

**Supplementary Information:**

The online version contains supplementary material available at 10.1186/s12885-025-14819-0.

## Introduction

Cancer immunotherapy has achieved remarkable success in recent years, especially with the advent of immune checkpoint inhibitors (ICIs), which enhance the body’s immune response against tumors and have demonstrated clinical efficacy across multiple cancer types [1]. Nevertheless, a substantial proportion of patients fail to respond to ICIs, largely due to immunosuppressive mechanisms within the tumor microenvironment (TME) [2]. The TME, comprising diverse immune cells, stromal components, and cytokines, plays a central role in shaping tumor progression and therapeutic response. Recent studies emphasize the importance of comprehensive molecular and immune profiling of the TME to guide personalized immunotherapy [3, 4]. For instance, predictive signatures derived from exhausted T cells [5] or plasma cells [3] have been proposed to stratify patients for ICI response across multiple tumor types. Moreover, systemic immune-inflammation indices such as the Systemic Immune-Inflammation Index (SII) have been shown to correlate with prognosis by reflecting the immunological status of the TME [6].

Hepatocellular carcinoma (HCC), accounting for the majority of primary liver cancers, exhibits high global incidence and mortality [7]. Its immune microenvironment is highly immunosuppressive, enabling tumor cells to escape immune surveillance via epithelial-mesenchymal transition (EMT) and immunosuppressive cytokine secretion [8]. Therefore, elucidating the immune evasion mechanisms in HCC is crucial for identifying novel immunotherapeutic targets and improving clinical outcomes.

Syntaxin-7 (STX7), a member of the SNARE family, mediates intracellular vesicle trafficking and membrane fusion [9]. STX7 is frequently upregulated in multiple malignancies and has been linked to tumor progression and adverse prognosis [10–12]. Mechanistically, STX7 promotes invadopodia formation during cancer invasion by modulating MT1-MMP trafficking [13]. Loss of STX7 impairs invadopodia formation and extracellular matrix degradation, underscoring its role in protease delivery [10]. Although emerging evidence implicates STX7 in immune regulation within the tumor microenvironment, its role in immune escape in HCC remains insufficiently characterized.

In this study, we performed a pan-cancer multi-omics analysis of STX7 using integrated datasets from tumor and normal tissues to elucidate its associations with expression patterns, clinical parameters, and patient prognosis. Particular attention was given to immune-related pathways, including TNF-NF-κB signaling, interferon-α/γ responses, and inflammatory processes. Single-cell and spatial transcriptomic analyses further revealed STX7 as a potential biomarker associated with macrophage infiltration. The functional role of STX7 in promoting HCC malignancy was subsequently validated through in vivo and in vitro experiments.

## Materials and methods

### Data sources

STX7 expression levels across multiple cancers, along with related clinical data, were obtained from The Cancer Genome Atlas (TCGA) and the Genotype-Tissue Expression (GTEx) databases via the UCSC Xena platform [14]. Single nucleotide variant (SNV) data for multiple cancer types, alongside methylation data from HM27 and HM450 arrays, were retrieved from the Cancer Genomics Portal (cBioPortal) [15]. Transcriptomic data were processed using a log2 transformation (per million transcripts (TPM) + 1). A detailed list of the 33 cancer types included in this analysis is provided in Table S1.

The HCC scRNA-seq dataset (GSE149614) and spatial transcriptomic data for one patient with HCC (GSM7661260 in GSE238264) were both retrieved from the GEO database.

### Pan-cancer analysis of differential STX7 expression, diagnosis, and prognosis

We integrated data from the TCGA and GTEx databases to examine STX7 mRNA expression levels in tumor and normal tissues across 33 cancer types. Differential gene expression was analyzed using the ‘ggplot2’ package in R, with violin plots illustrating expression differences across various cancer subtypes. Additionally, the ‘pROC’ package in R was used to generate receiver operating characteristic (ROC) curves for the cancer types of interest, evaluating the diagnostic value of STX7 expression. To evaluate the prognostic significance of STX7 expression levels, Kaplan-Meier survival analysis and univariate Cox regression were performed using the ‘survival’ package. These analyses aimed to determine the impact of high versus low STX7 expression on overall survival (OS), disease-specific survival (DSS), progression-free survival (PFS), and disease-free survival (DFS).

### Differential gene expression in high and low STX7 subgroups

To assess gene expression differences between high and low STX7 subgroups in each cancer type, patients were ranked according to STX7 mRNA expression levels. The top 30% of patients were classified as the high STX7 subgroup, and the bottom 30% as the low STX7 subgroup. Differential expression analysis was conducted using the ‘limma’ R package, which calculates the log2 (fold change) and adjusted *p-values* for each gene across various cancer types. Genes with adjusted *p-values < 0.05* were considered differentially expressed genes (DEGs). The detailed DEG results for the high and low STX7 subgroups across different cancer types are provided in Table S2.

### Gene set enrichment analysis (GSEA)

A ‘gmt’ file containing 50 hallmark gene sets (h.all.v7.4.symbols.gmt) was downloaded from the Molecular Signatures Database (MSigDB, https://www.gsea-msigdb.org/gsea/index.jsp) [16]. This file was used to calculate the normalized enrichment scores (NES) and false discovery rates (FDR) for high and low STX7 expression groups across various biological processes in each cancer type. Gene set enrichment analysis (GSEA) was performed using the ‘clusterProfiler’ R package. The results were visualized in bubble plots generated using the ‘ggplot2’ R package.

### Immunological function of STX7 in the pan-cancer microenvironment and its consequences

To investigate the involvement of STX7 in immune cell infiltration across various cancers, the ‘ESTIMATE’ R package (version 1.0.13) [17] was used to calculate the ESTIMATE, stromal, and immune scores for tumor samples. Immune checkpoint markers from previous studies [18] were collected, and their association with STX7 expression levels was assessed. Using the subtype feature of the TISIDB database (http://cis.hku.hk/TISIDB/), heatmaps were generated through the Chemokine and Immunomodulator modules to visualize the relationship between STX7 expression and chemokines, chemokine receptors, and immune-stimulatory elements. The relationship between STX7 and immune cell infiltration was analyzed using the single-sample Gene Set Enrichment Analysis (ssGSEA) algorithm [19].

### Single-cell RNA sequencing data processing

It encompasses data from 10 primary tumor (PT) patients, 2 portal vein tumor thrombus (PVTT) patients, 1 metastatic lymph node (MLN) patient, and 8 normal liver tissue (NLT) patients (Table S3). The raw dataset comprises 25,479 genes and 71,915 cells. Data filtering was performed using the PercentageFeatureSet function to calculate the proportion of mitochondrial and ribosomal RNA genes. Cells were selected based on the following criteria: [1] unique molecular identifier (UMI) count > 500; [2] gene count per cell between 500 and 8,000; [3] mitochondrial gene proportion < 30%; and [4] ribosomal gene proportion < 3%. After applying these filters, a total of 71,547 cells were retained for further analysis.

Dimensionality reduction and clustering analyses were performed using the ‘Seurat’ R package [20]. The filtered data were initially normalized using the Log-Normalize and ScaleData functions. To identify genes with high variability, the FindVariableFeatures function was applied. Principal component analysis (PCA) was performed for dimensionality reduction, selecting the top 15 principal components for further investigation. Batch effects were mitigated using the ‘Harmony’ package, and Uniform Manifold Approximation and Projection (UMAP) was employed for visualization. Clustering of the data was performed using the FindClusters function.

For pseudotime analysis of monocytes and macrophages, the ‘monocle3’ R package [21] was used. Initially, monocyte-macrophage data were extracted from the Seurat object, followed by dimensionality reduction and clustering analysis. The Seurat object was converted into a ‘monocle3’ object, and the orderCells function was used to arrange the cells along their developmental trajectory, reflecting their temporal progression. The results were visualized using R packages such as ‘monocle3,’ ‘plot1cell,’ and ‘ggplot2.’

### Cell communication analysis

Cell-cell communication analysis was conducted using the ‘CellChat’ R package [22]. Initially, the annotated Seurat object was converted into a CellChat object, and cells were classified based on high or low STX7 expression levels. Subsequently, functional identification methods, such as identifying overexpressed genes, were applied to pinpoint genes enriched in specific cell types. This approach enabled a more in-depth exploration of the cell-cell communication pathways involved.

### Spatial transcriptome data analysis

Spatial transcriptome analysis was performed on the ST data using the Seurat package, following a similar approach to scRNA-seq analysis.

### Experimental methods

Detailed information on the experimental methods can be found in Supplementary Material 2.

### Statistical analysis

To compare differences between two groups of normally distributed variables, a Student’s t-test was applied. For comparisons across multiple groups, one-way analysis of variance (ANOVA) was performed. In cases where variables were not normally distributed, the Wilcoxon test was employed for two-group comparisons, and the Kruskal-Wallis test was used for multiple group comparisons. Pearson’s correlation was applied to calculate correlation coefficients, and heatmaps were generated using R packages. A *p-value* of less than 0.05 was considered statistically significant.

## Results

### Pan-cancer analysis of STX7 expression and genomic alterations

**Fig. 1:**
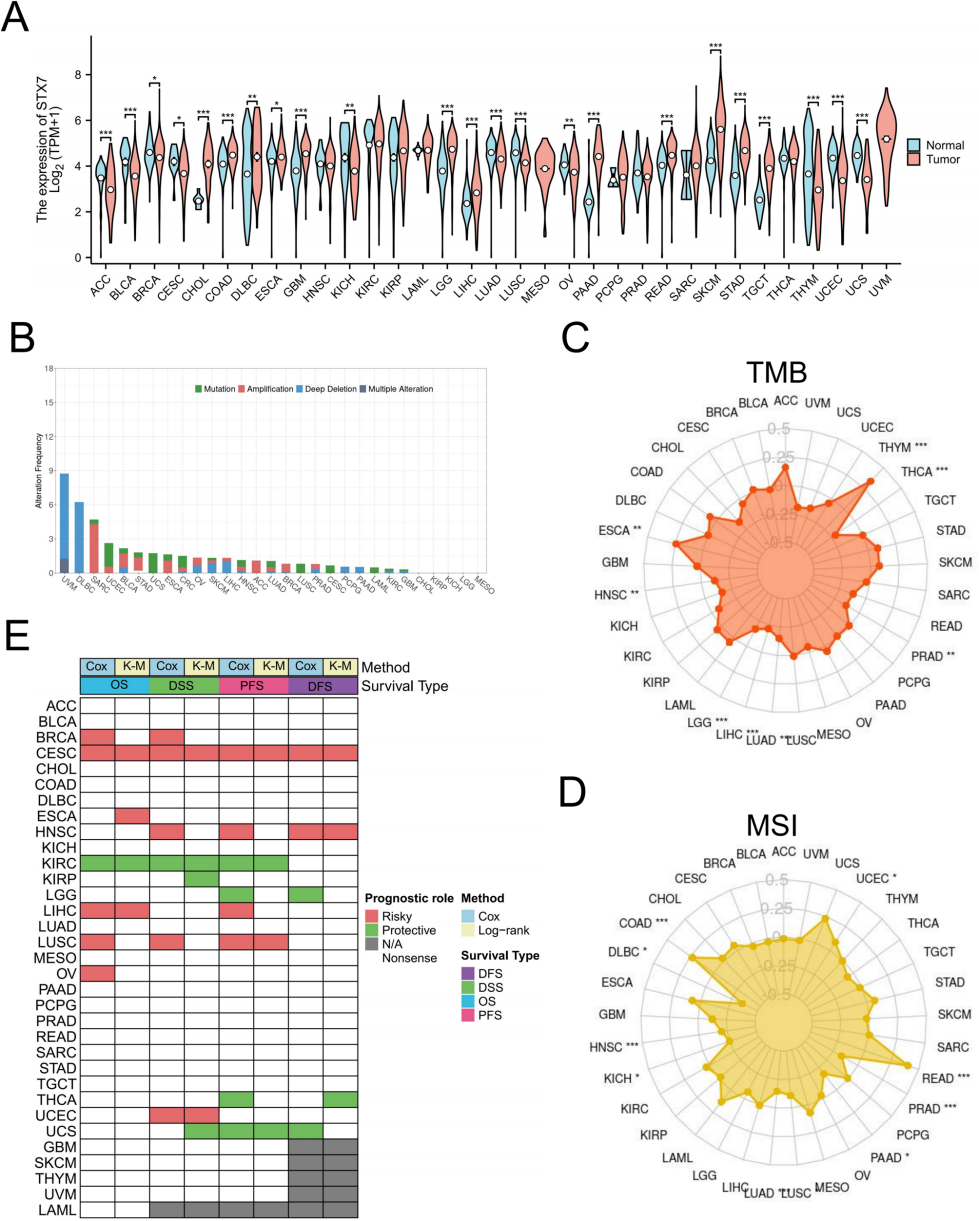
Pan-cancer analysis of STX7 expression, genomic alterations, and prognostic significance. (**A**)Integrated analysis of STX7 expression in tumor and healthy tissue samples was conducted using the TCGA and GTEx datasets. (**B**) Pan-cancer investigation of genomic alterations in STX7 was performed using data from the TCGA database, examining mutations, amplifications, and deep deletions. Radar plots were utilized to represent the correlation between STX7 and tumor mutational burden (TMB) (**C**) as well as microsatellite instability (MSI) (**D**). (**E**) A heatmap was generated to show the association between STX7 expression and overall survival (OS), disease-specific survival (DSS), disease-free survival (DFS), and progression-free survival (PFS)

A comprehensive analysis of STX7 mRNA expression across cancer types was performed using data from the TCGA and GTEx databases. This analysis revealed significant alterations in STX7 expression across 23 cancer types (Fig. 1A). Additionally, we examined potential genomic alterations in STX7 across specific cancers. The findings showed that STX7 amplification was most common in SARC, while deep deletions were frequent in UVM and DLBC (Fig. 1B). STX7 expression was positively correlated with tumor mutational burden (TMB) in ESCA and THYM (Fig. 1C) and with microsatellite instability (MSI) in COAD, READ, and UCEC (Fig. 1D). These results suggest a significant association between STX7 expression and genomic instability across various cancer types.

### Diagnostic and prognostic value of STX7 in multiple cancers

The ROC curve indicates that STX7 could serve as a potential diagnostic biomarker for certain cancers (Figure S1). To further investigate its prognostic value, we analyzed 33 cancer types from the TCGA database and examined the correlation between STX7 expression and various survival outcomes, including OS, DSS, PFS, and DFS. Univariate Cox regression analysis showed that high STX7 expression was significantly associated with poor OS in BRCA, CESC, LIHC, LUSC, and OV, while acting as a protective factor in KIRC. For DSS, STX7 was a risk factor for BRCA, CESC, HNSC, LUSC, and UCEC, and a protective factor in KIRC. For DFS, STX7 was a risk factor in CESC and HNSC, but a protective factor in LGG and UCS. For PFS, STX7 was a risk factor for CESC, HNSC, LUSC, and LIHC, and a protective factor in KIRC, THCA, LGG, and UCS. Kaplan-Meier survival analysis confirmed these findings (Fig. 1E). In summary, elevated STX7 expression was generally associated with poorer prognosis in patients with BRCA, CESC, LIHC, LUSC, HNSC, and UCEC.

### GSEA of STX7 in pan-cancer

**Fig. 2:**
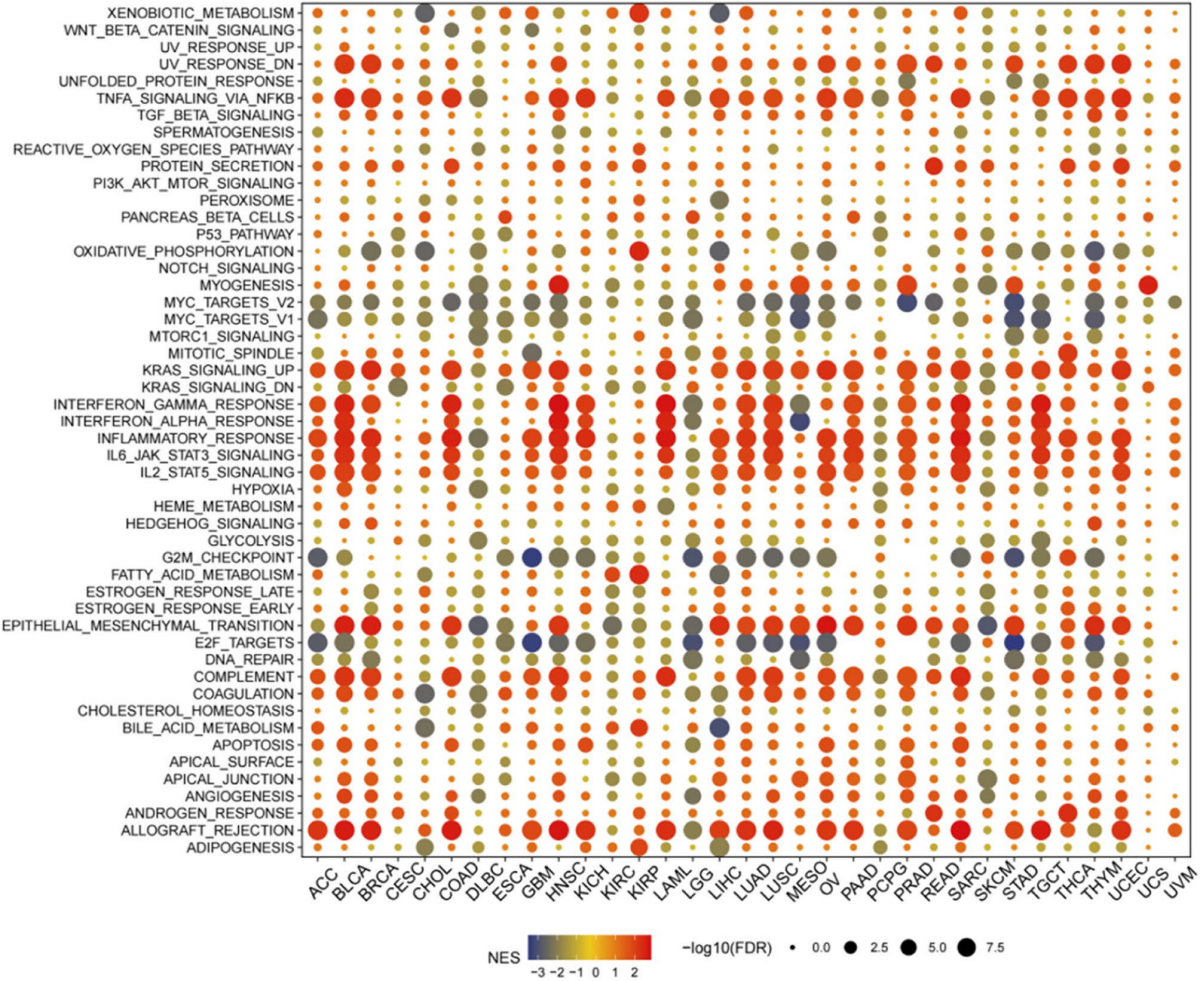
Hallmark gene set enrichment analysis (GSEA) of STX7 in pan-Cancer. This analysis demonstrates the enrichment of hallmark gene sets associated with STX7 across different cancer types. The circle size reflects the false discovery rate (FDR) for each enriched term in the respective cancers, and the color represents the normalized enrichment score (NES) for each term

To identify cancer-related markers linked to STX7, we conducted GSEA on DEGs between the low and high STX7 subgroups in each cancer type. STX7 expression was significantly associated with immune-related pathways, including TNF-NFκB signaling, IFN-α and IFN-γ responses, inflammatory response, and allograft rejection pathways, particularly in BLCA, BRCA, COAD, HNSC, KICH, LAML, LUAD, LUSC, and SARC. These results imply a potential link between STX7 expression and immune activation within the tumor microenvironment (TME). Moreover, in the high STX7 subgroup of BLCA, BRCA, COAD, HNSC, LIHC, LUAD, LUSC, MESO, OV, PAAD, PRAD, READ, SARC, STAD, THYM, and UCEC, markers of epithelial-mesenchymal transition (EMT) were significantly enriched. suggesting that STX7 may contribute to cancer progression via EMT. Furthermore, the KRAS signaling pathway, IL6/JAK/STAT3 pathway, and IL-2/STAT5 pathway were strongly linked to STX7 expression in cancer. These findings underscore the potential of STX7 as a key player in immune activation and cancer progression, providing new avenues for further investigation (Fig. 2).

### Role of STX7 in cancer immune infiltration

**Fig. 3:**
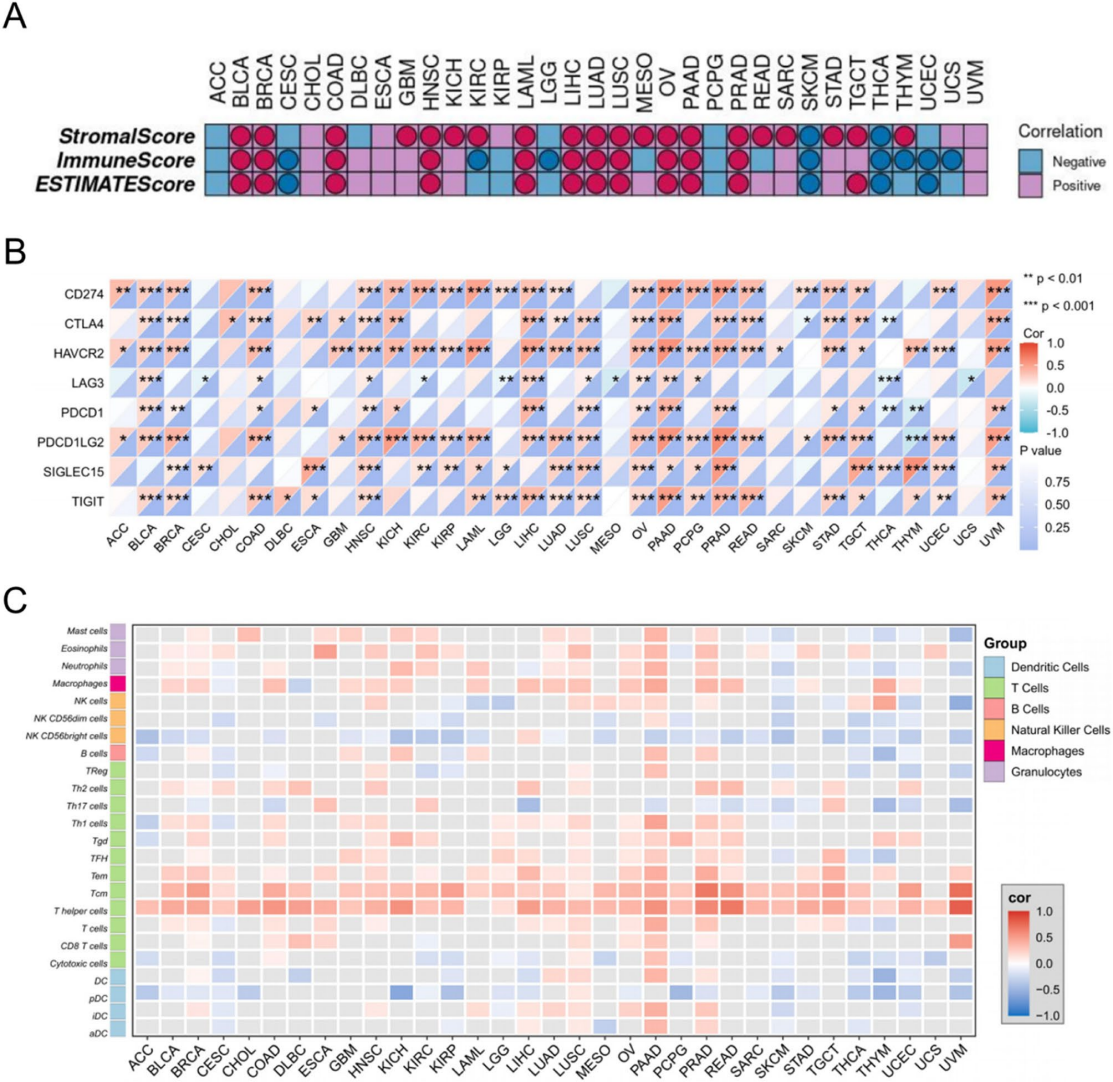
Correlation of STX7 with immune infiltration. **A** A heatmap shows the relationships between STX7 expression and ESTIMATEScore, ImmuneScore, and StromalScore, with circles marking statistically significant correlations (*p* < 0.05). **B** A heatmap displays the associations between immune checkpoint markers and STX7 expression across multiple cancer types. Statistical significance is denoted by *, **, and *** for *p-*values* < 0.05*,* < 0.01*,* and < 0.001*, respectively. **C **The ssGSEA analysis reveals immune cell infiltration patterns across pan-cancer

To investigate the immunological role of STX7 within the cancer microenvironment, we calculated the ESTIMATE score for STX7 across a range of cancer types. As shown in Fig. 3A, a positive correlation was observed between STX7 and the ESTIMATEScore, StromalScore, and ImmuneScore across several cancer types, including those from TCGA, such as BLCA, BRCA, COAD, HNSC, LAML, LIHC, LUAD, LUSC, OV, PAAD, and PRAD. However, in certain cancers, such as SKCM and THCA, STX7 exhibited a negative correlation with these scores. Additionally, cancers exhibiting a positive correlation between STX7 and ImmuneScore also showed a positive association with most immune checkpoint genes (Fig. 3B). Notably, genes such as HAVCR2, PDCD1LG2, CD274, and TIGIT exhibited the strongest positive correlations with STX7 across more than 20 cancer types, suggesting a role for STX7 in immune checkpoint-mediated regulation.

We also explored the association between STX7 and various chemokines, receptors, and immune stimulators using TISIDB. The heatmap in Figure S2 shows that STX7 is positively associated with various chemokines, immune receptors, stimulatory factors, and elevated promoter methylation across several cancers. This indicates that STX7 expression modulates the immune microenvironment via chemokine signaling, likely contributing to tumor progression by altering the immune landscape. Additionally, we assessed STX7 expression across various immune cell types. Using the ssGSEA algorithm, we found a significant correlation between STX7 and 24 immune cell types, underscoring its potential role in shaping the immune profile within cancerous environments (Fig. 3C).

### Single-cell RNA sequencing analysis in HCC

**Fig. 4:**
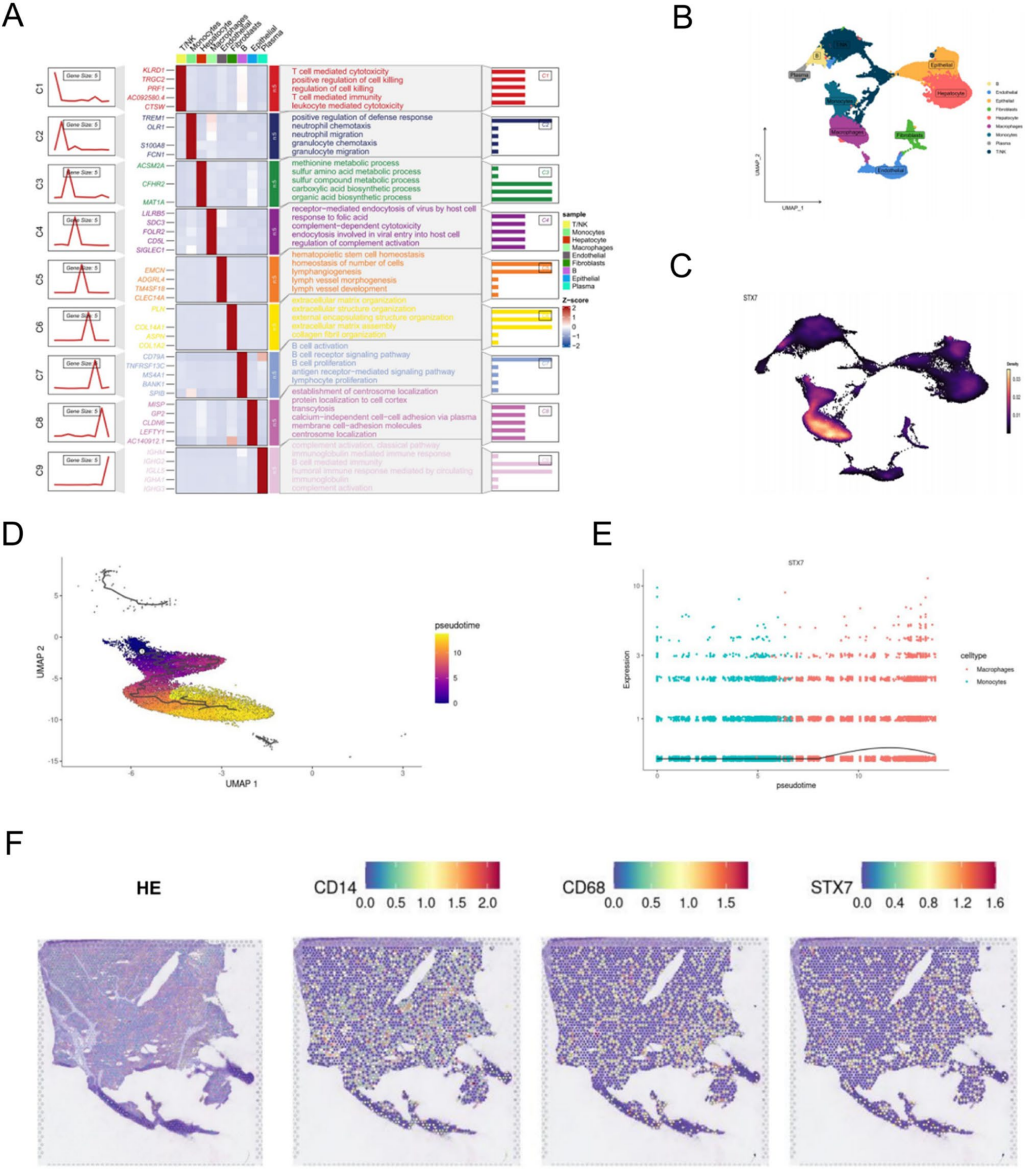
Single-cell and spatial transcriptomic analysis of STX7 expression in HCC. **A **Heatmaps of the top five genes and functional enrichment analysis for different cell types in single-cell transcriptome. **B **The UMAP plot illustrates the distribution of distinct cell subtypes within the multicellular environment of HCC patients, with each subtype represented by a unique color. **C** The expression levels of STX7 in HCC cells. **D** Pseudotime and trajectory analysis of macrophages and monocytes in HCC. (**E**) The trend of STX7 expression across pseudotime variation. **F** Spatial transcriptomic sections showing overlapping patterns of H&E staining, CD14, CD68, and STX7 expression

We included 10 HCC patients with varying tumor-node-metastasis (TNM) stages and hepatitis virus infection statuses. Eighteen distinct cell clusters were identified and annotated based on classical cell-type marker genes, resulting in categories including hepatocytes, endothelial cells, T/NK cells, epithelial cells, macrophages, B cells, fibroblasts, monocytes, and plasma cells (Figs. 4A, B).

### Elevated STX7 expression in macrophage cells in HCC

In HCC, STX7 exhibits a predominant expression pattern in monocytes and macrophages, particularly in macrophages (Fig. 4C). Pseudotime trajectory analysis further demonstrates the variability in STX7 expression during the progression of the monocyte-macrophage lineage (Figs. 4D, E).

Spatial transcriptomic data confirmed that STX7 expression patterns overlapped substantially with those of the macrophage marker CD14 and CD68 in HCC (Fig. 4F), implied potential co-localization of these genes.

### Intercellular communication in macrophages with varying STX7 expression levels

**Fig. 5:**
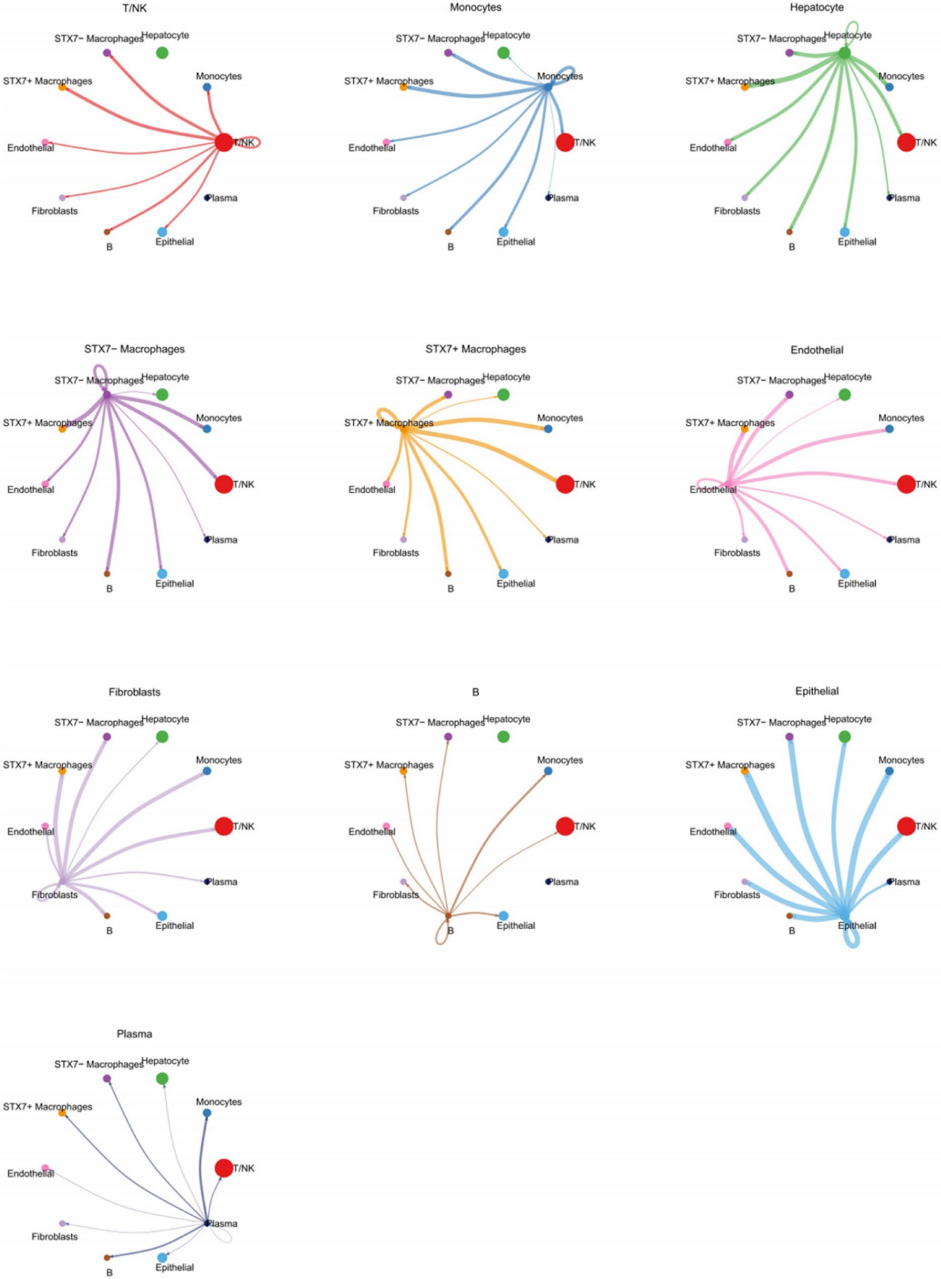
Circle plots showing cell-cell communications of main cell clusters. Each cell cluster acts as a signaling sender or signaling receiver conducting intercellular crosstalk with STX7 + macrophages and STX7- macrophages, respectively

**Fig. 6:**
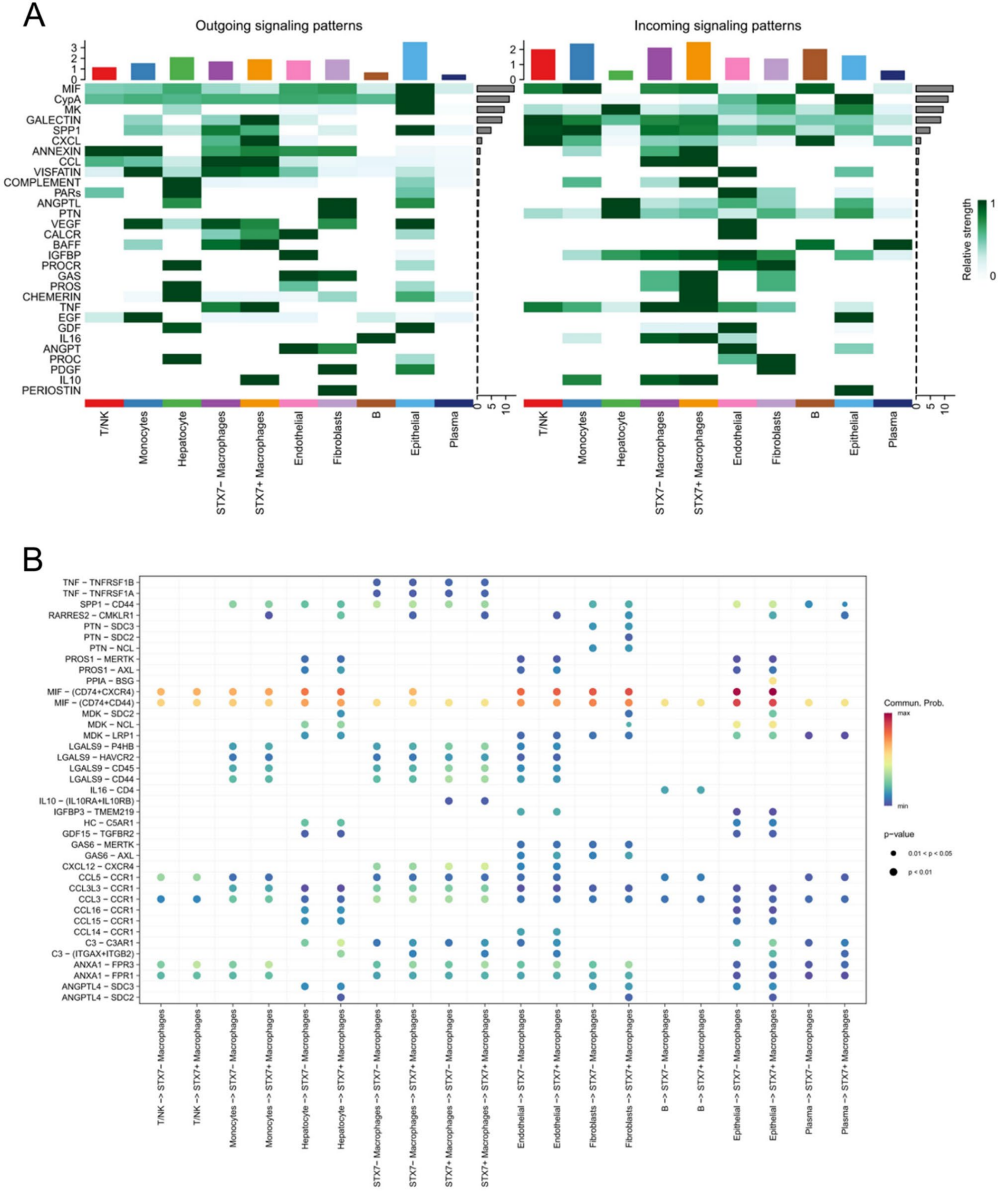
Regulatory role of STX7 in cell-cell communication. **A** Overview of incoming and outgoing information flows mediated by various signaling pathways in the main cell clusters. **B** Communication probabilities of key ligand-receptor pairs that mediate cell-cell interactions between main cell clusters and STX7 + or STX7- macrophages. The color of each dot indicates the probability of communication, while the size of the dot corresponds to the *p-*value. A lack of corresponding ligand-receptor pairs is indicated by empty meaning, suggesting no communication in that cell

To investigate the regulatory role of STX7 in intercellular communication, we compared STX7 + and STX7- macrophages and used the CellChat tool to analyze differences in signaling interactions. The analysis revealed that STX7 + macrophages, as receivers, showed significantly stronger communication with epithelial cells and fibroblasts than STX7- macrophages. Additionally, as signal senders, STX7 + macrophages showed enhanced interactions with monocytes and T/NK cells, compared to STX7- macrophages (Fig. 5). In several signaling pathways, STX7 + macrophages demonstrated stronger overall signal transmission than STX7- macrophages. These pathways included input signals (ANNEXIN, COMPLEMENT, GAS, PROS, CHEMERIN, IL16) and output signals (GALECTIN, CXCL, BAFF, TNF, IL10) (Fig. 6A). STX7 + macrophages exhibited prominent activity in ligand-receptor (L-R) interactions with endothelial cells, monocytes, and fibroblasts. Specifically, interactions with monocytes and fibroblasts prominently activated the MIF (CD74 + CXCR4) signaling pathway, while communication with endothelial cells resulted in the activation of the C3 (ITGAX + ITGB2) and RARRES2 (CMKLR1) signaling pathways (Fig. 6B). These findings underscore the critical role of STX7 + macrophages in orchestrating intercellular signaling within the immune microenvironment.

### STX7 enhances the progression of hepatocellular carcinoma and modulates macrophage recruitment

**Fig. 7:**
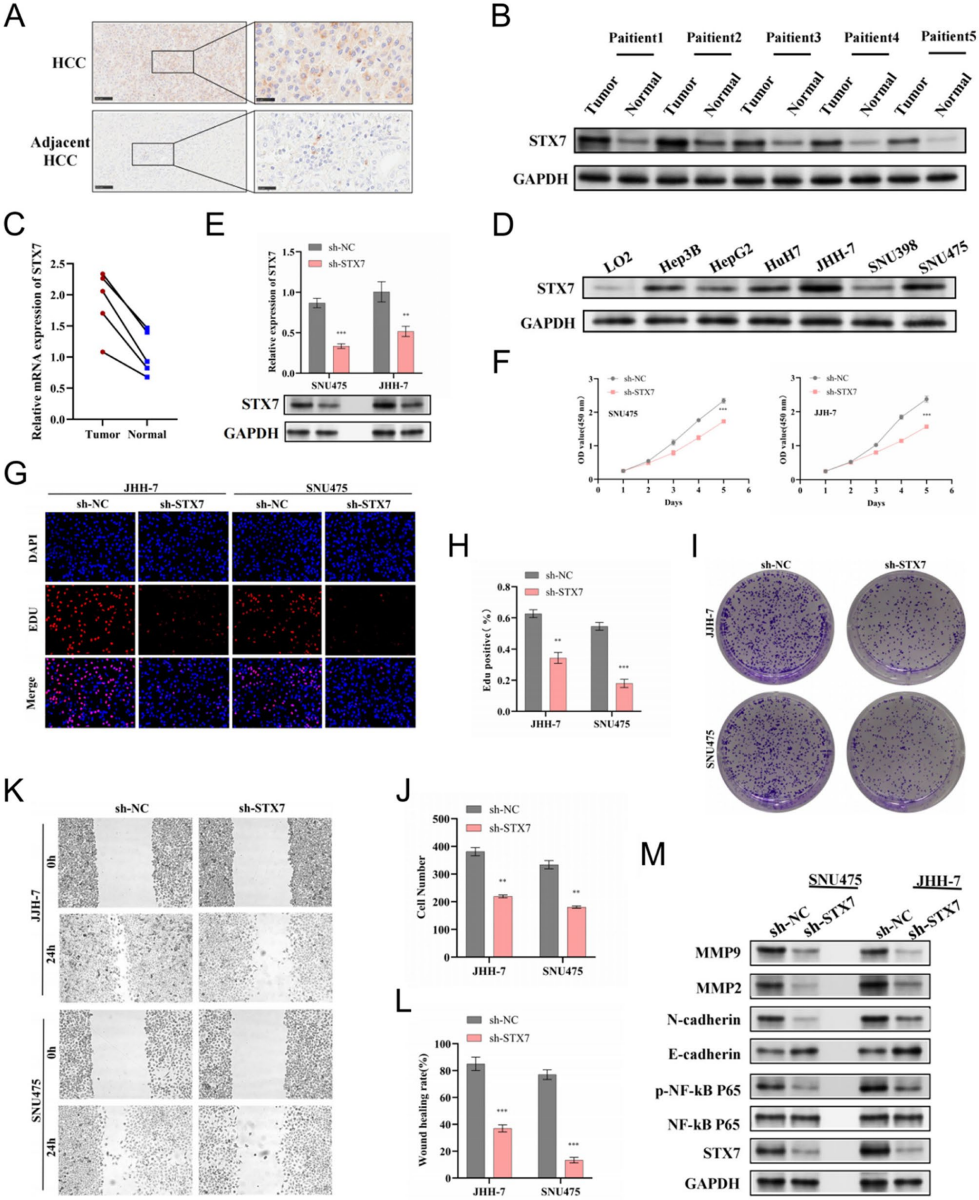
Evaluation of the role of STX7 in regulating the malignancy of hepatocellular carcinoma cells through the NF-κB pathway. STX7 expression in paired HCC tumor and adjacent non-cancerous tissues, as assessed by immunohistochemical staining (**A**), western blot (**B**), and qPCR (**C**) analysis, respectively. **D** Expression levels of STX7 protein in liver cell lines. **E** mRNA and protein levels of STX7 in transfected cells. **F-J** The effect of STX7 knockdown on tumor cell proliferation, assessed using CCK-8, EdU uptake assays, and colony formation assays. (**K**,** L**) Tumor cell migration following STX7 knockdown, assessed through a wound healing assay. **M** Western blot results showed the effects of STX7 knockdown on EMT-and NF-κB-related proteins in JHH-7 and SNU-475 cells

Through comprehensive experimental analyses, we confirmed the upregulation of STX7 protein expression in HCC tissues (Fig. 7A-C). Compared to the relatively low expression observed in L-02 cells, STX7 was markedly upregulated in the HCC cell lines (Fig. 7D). The full-length, uncropped Western blot images supporting this result are provided in the supplementary file titled *“Uncropped_FullLength_Original_WesternBlots”*. Leveraging the naturally high STX7 expression in these HCC cell lines, stable knockdown of STX7 was established in JHH-7 and SNU-475 cells (Fig. 7E). STX7 silencing significantly inhibited HCC cell proliferation, as shown by the CCK-8, EdU, and colony formation assay (Figs. 7F-J). Scratch wound assays further revealed that STX7 knockdown impaired cell migration and invasion (Figs. 7K, L).

In JHH-7 and SNU-475 cells, STX7 protein downregulation resulted in a marked upregulation of E-cadherin levels and a simultaneous decrease in the expression of N-cadherin, MMP2, and MMP9. Additionally, p-NF-κB p65 expression was significantly reduced following STX7 knockdown, whereas the expression of NF-κB p65 remained largely unchanged. These findings suggest that STX7 may promote the epithelial-mesenchymal transition (EMT) process in HCC cells via the activation of the NF-κB signaling pathway (Figs. 7M). These full-length, uncropped Western blot images are provided in the supplementary file titled *“Uncropped_FullLength_Original_WesternBlots”*.

**Fig. 8:**
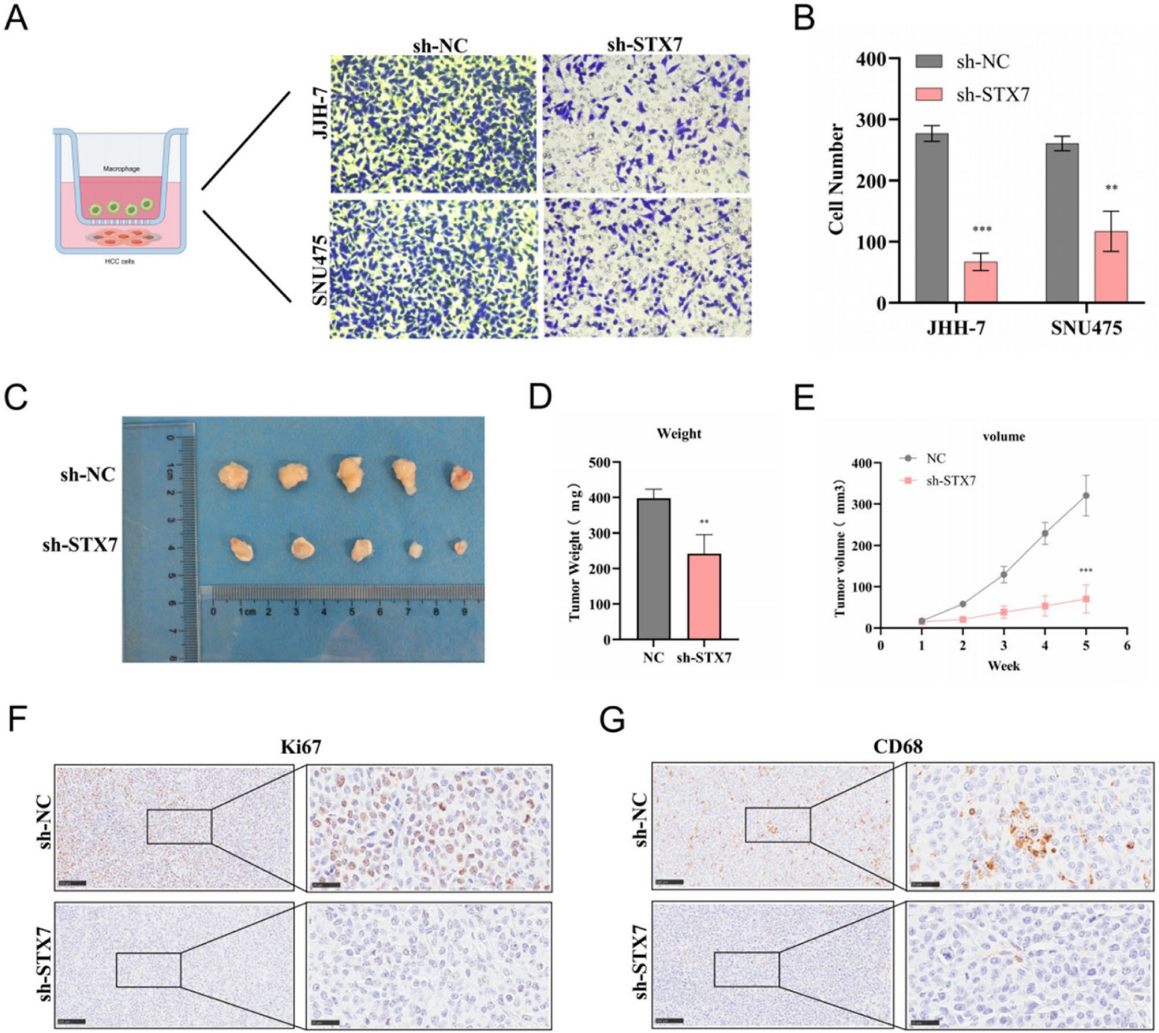
Investigating STX7-mediated macrophage infiltration and its impact on tumor progression in vivo. **A**, **B** Migration of macrophages toward tumor cells following STX7 knockdown in JHH-7 and SNU-475 cells. **C** Representative images of tumors. **D** Tumor weights. **E** Tumor volumes. **F**, **G** Immunohistochemical staining of tumor tissues for CD68 and Ki-67. Statistical significance is represented as *P < 0.05, **P < 0.01, ***P < 0.001

STX7 knockdown also led to a substantial reduction in macrophage infiltration (Figs. 8A, B). In vivo experiments demonstrated that STX7 silencing significantly reduced subcutaneous xenograft tumor growth and macrophage infiltration compared to controls (Figs. 8C-G). These findings highlight the critical role of STX7 in promoting HCC cell proliferation, migration, and invasion.

## Discussion

STX7 has garnered increasing attention due to its involvement in cellular processes such as vesicle trafficking, autophagy, and immune regulation [23]. While its physiological role is well established, recent evidence suggests that STX7 dysregulation may contribute to tumorigenesis and immune remodeling in a cancer-type-specific manner [10, 11, 24, 25]. In the context of tumor microenvironment (TME)--driven progression, several studies have highlighted the need for integrative multi-omics and machine learning approaches to uncover immune-associated molecular drivers [4]. Building on these findings, we conducted a pan-cancer analysis of STX7 to clarify its expression patterns, prognostic associations, functional enrichment, and immune infiltration characteristics, aiming to evaluate its potential as a biomarker and immunotherapy target.

STX7 expression is elevated in multiple malignancies, including CHOL, COAD, LIHC, READ, and STAD, and is often linked to poor prognosis through its influence on immune modulation within the TME [10, 12]. In contrast, high STX7 expression in renal tumors such as KIRC and KIRP is associated with better outcomes, suggesting a context-dependent function. This prognostic divergence may be attributed to differences in tumor cell biology, tissue origin, or microenvironmental context. These findings highlight the need to interpret STX7’s prognostic significance within the specific molecular and clinical context of each cancer type.

Gene set enrichment analysis (GSEA) revealed that high STX7 expression is enriched in immune-related pathways such as TNF-NF-κB signaling, IFN-α and IFN-γ responses, inflammatory response, and allograft rejection, indicating its broad involvement in immune regulation. These pathways imply that STX7 may orchestrate both immune activation and suppression, thereby altering the equilibrium between tumor immune surveillance and immune escape [26]. Specifically, TNF-NF-κB signaling can activate tumor-associated macrophages (TAMs), induce M2 polarization, and foster an immunosuppressive TME that facilitates tumor proliferation, angiogenesis, and distant metastasis [4, 27]. NF-κB also regulates epithelial-mesenchymal transition (EMT) via transcriptional activation of Snail, Twist, and ZEB1, thus accelerating tumor invasion and dissemination [28]. Our in vitro assays further confirmed that STX7 promotes EMT in HCC cells via NF-κB signaling, enhancing cellular invasiveness and metastatic capacity. Collectively, these results highlight STX7 as a potential therapeutic node in NF-κB signaling, offering opportunities to attenuate tumor-promoting inflammation and reinvigorate anti-tumor immune responses.

High STX7 expression, along with elevated ImmuneScores, correlates with increased expression of immune checkpoint genes such as PD-L1, TIGIT, and HAVCR2, suggesting that STX7 may contribute to immune evasion through inhibitory signaling pathways [29]. Single-cell RNA-seq and spatial transcriptomic analyses further revealed that STX7 is predominantly expressed in macrophages within the HCC microenvironment, likely corresponding to tumor-associated macrophages (TAMs). These findings prompted the hypothesis that STX7 may regulate macrophage behavior and function within the tumor microenvironment.

Our single-cell and spatial transcriptomic analyses revealed that STX7 is predominantly expressed in monocyte-derived macrophages in HCC, with dynamic upregulation during differentiation and co-localization with CD14 and CD68 in tumor regions. These STX7⁺ macrophages exhibited enhanced intercellular communication with epithelial, stromal, and immune cells, and engaged in immunoregulatory ligand–receptor interactions such as MIF–CD74–CXCR4, RARRES2–CMKLR1, and C3–ITGAX/ITGB2 [30–32]. They also secreted elevated levels of IL10, TNF, and GALECTIN, suggesting a hybrid immunosuppressive and pro-angiogenic phenotype [33–35]. This is consistent with the established roles of TAMs in promoting tumor progression through immune evasion, angiogenesis, and extracellular matrix remodeling via pathways like CSF1–CSF1R, CCL2–CCR2, and VEGF–VEGFR [36, 37]. Collectively, these findings highlight STX7 as a potential immunomodulatory hub, meriting further mechanistic validation and exploration as a candidate target for macrophage-focused immunotherapy.

However, several limitations of this study should be acknowledged. Due to sample availability, qRT-PCR and Western blot validations were conducted on five paired HCC and adjacent tissues, which may limit the generalizability of the findings. Although subcutaneous xenograft models were used to assess tumor growth, the absence of orthotopic liver and lung metastasis models may limit physiological relevance, and future studies will incorporate these models to better evaluate the in situ and metastatic roles of STX7. Furthermore, the functional heterogeneity between STX7⁺ and STX7⁻ macrophages was not experimentally dissected, which constrains the depth of mechanistic insights. Future studies will address these limitations to better elucidate the mechanistic role of STX7 in HCC progression and metastasis.

## Conclusion

In conclusion, STX7 is overexpressed in tumor tissue, associated with poor prognosis, and plays a role in immune regulation, evasion, and tumor progression. Both in vitro and in vivo studies confirmed STX7 knockout significantly reduced HCC proliferation, migration, and macrophage infiltration, while also inhibiting EMT through NF-κB signaling. Nonetheless, our understanding of the link between STX7 and cancer progression remains limited, necessitating further studies to elucidate its mechanisms, functions, and therapeutic applications.

## Supplementary Information

Supplementary Material 1.

Supplementary Material 2.

Supplementary Material 3.

Supplementary Material 4.

Supplementary Material 5.

Supplementary Material 6.

Supplementary Material 7.

Supplementary Material 8.

## Data Availability

The original data pertaining to this research are included within the article and its supplementary materials. For any further inquiries, please contact the corresponding author.
