# Supplementary figures and images for: Syntaxin-7 promotes EMT and tumor progression via NF-κB signaling and is associated with macrophage infiltration: pan-cancer analysis and experimental validation in hepatocellular carcinoma
Source: BMC Cancer. 2025 Sep 25;25:1430. doi: 10.1186/s12885-025-14819-0 (PMC12465986; doi:10.1186/s12885-025-14819-0)

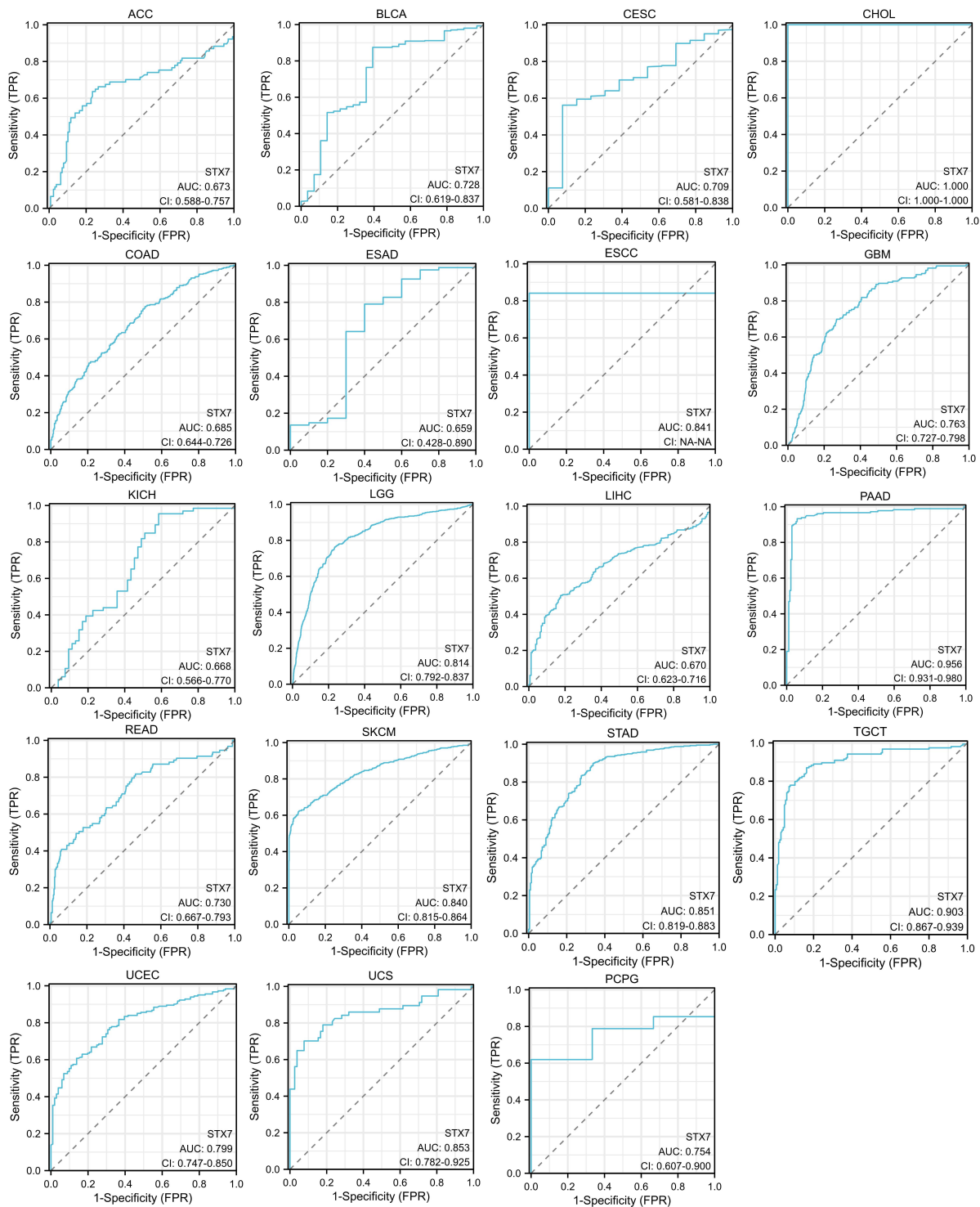

Supplement: Supplementary file 1 — Supplementary Material 1. [file 12885_2025_14819_MOESM1_ESM.pdf]
