# Supplementary material for: Syntaxin-7 promotes EMT and tumor progression via NF-κB signaling and is associated with macrophage infiltration: pan-cancer analysis and experimental validation in hepatocellular carcinoma
Source: BMC Cancer. 2025 Sep 25;25:1430. doi: 10.1186/s12885-025-14819-0 (PMC12465986; doi:10.1186/s12885-025-14819-0)
Supplement: Supplementary file 2 — Supplementary Material 2. [file 12885_2025_14819_MOESM2_ESM.pdf]

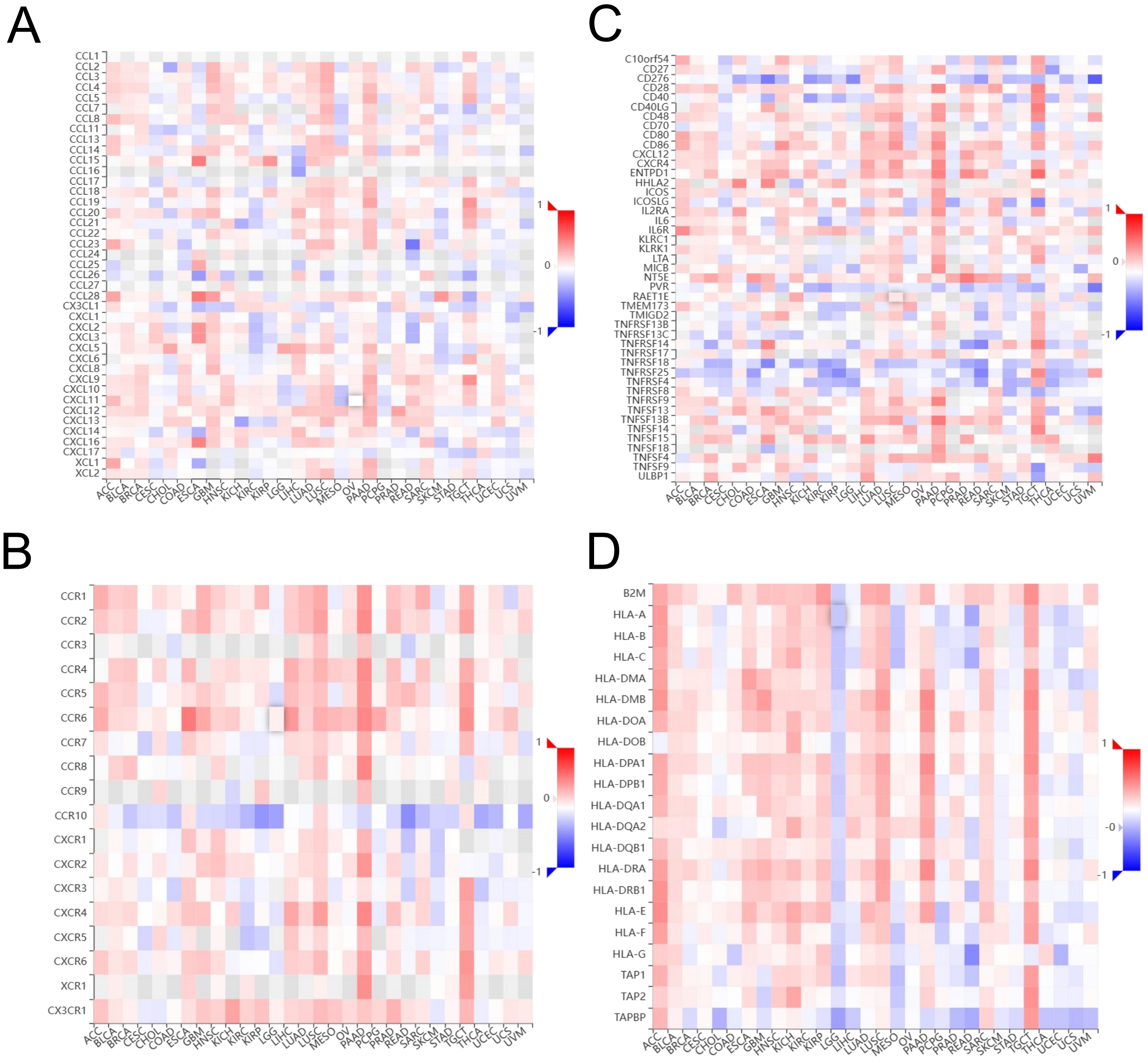
A
B
C
D
CCL1
CCL2
CCL3
CCL4
CCL5
CCL7
CCL8
CCL11
CCL13
CCL14
CCL15
CCL16
CCL17
CCL18
CCL19
CCL20
CCL21
CCL22
CCL23
CCL24
CCL25
CCL26
CCL27
CCL28
CX3CL1
CXCL1
CXCL2
CXCL3
CXCL5
CXCL6
CXCL8
CXCL9
CXCL10
CXCL11
CXCL12
CXCL13
CXCL14
CXCL16
CXCL17
XCL1
XCL2
CCR1
CCR2
CCR3
CCR4
CCR5
CCR6
CCR7
CCR8
CCR9
CCR10
CXCR1
CXCR2
CXCR3
CXCR4
CXCR5
CXCR6
XCR1
CX3CR1
C10orf54
CD27
CD276
CD28
CD40
CD40LG
CD48
CD70
CD80
CD86
CXCL12
CXCR4
ENTPD1
HHLA2
ICOS
ICOSLG
IL2RA
IL6
IL6R
KLRC1
KLRK1
LTA
MICB
NT5E
PVR
RAET1E
TMEM173
TMIGD2
TNFRSF13B
TNFRSF13C
TNFRSF14
TNFRSF17
TNFRSF18
TNFRSF25
TNFRSF4
TNFRSF8
TNFRSF9
TNFSF13
TNFSF13B
TNFSF14
TNFSF15
TNFSF18
TNFSF4
TNFSF9
ULBP1
B2M
HLA-A
HLA-B
HLA-C
HLA-DMA
HLA-DMB
HLA-DOA
HLA-DOB
HLA-DPA1
HLA-DPB1
HLA-DQA1
HLA-DQA2
HLA-DQB1
HLA-DRA
HLA-DRB1
HLA-E
HLA-F
HLA-G
TAP1
TAP2
TAPBP
ACC BLCA BRCA CESC CHOL COAD ESCA GBM HNSC KICH KIRC KIRP LGG LIHC LUAD LUSC MESO OV PAAD PCPG PRAD READ SARC SKCM STAD TGCT THCA UCEC UCS UVM
1
0
-0
-1
