# Supplementary material for: Syntaxin-7 promotes EMT and tumor progression via NF-κB signaling and is associated with macrophage infiltration: pan-cancer analysis and experimental validation in hepatocellular carcinoma
Source: BMC Cancer. 2025 Sep 25;25:1430. doi: 10.1186/s12885-025-14819-0 (PMC12465986; doi:10.1186/s12885-025-14819-0)
Supplement: Supplementary file 3 — Supplementary Material 3. [file 12885_2025_14819_MOESM3_ESM.docx]

## Figure legend

**Figure S1** Pan-cancer diagnostic ROC curves for STX7..

**Figure S2** Heatmaps show correlations between STX7 expression and chemokines (A), receptors (B), and immunostimulators (C), as well as the relationship between STX7 promoter methylation levels and immunostimulators (D).
