# Supplementary material for: Syntaxin-7 promotes EMT and tumor progression via NF-κB signaling and is associated with macrophage infiltration: pan-cancer analysis and experimental validation in hepatocellular carcinoma
Source: BMC Cancer. 2025 Sep 25;25:1430. doi: 10.1186/s12885-025-14819-0 (PMC12465986; doi:10.1186/s12885-025-14819-0)
Supplement: Supplementary file 4 — Supplementary Material 4. [file 12885_2025_14819_MOESM4_ESM.docx]

# Supplementary Material 2

## Experimental methods

### Human clinical specimens

Human HCC tissues and paired adjacent non-tumor tissues were collected from 10 patients at the Guangxi Zhuang Autonomous Region People's Hospital. The adjacent tissues were located at least 2 cm away from the paired HCC tissues. Written informed consent was obtained from all patients. The study was approved by the Medical Ethics Committee of Guangxi Zhuang Autonomous Region People's Hospital (KY-GZR-2024-093).

### Immunohistochemical staining

The slides were hydrated and then treated with citrate buffer (pH 6.0) at 100°C for 10 minutes. Endogenous peroxidase activity was blocked with 3% H₂O₂ at room temperature for 15 minutes.Next, the slides were blocked with goat serum at 37°C for 1 hour and incubated overnight at 4°C with the specified primary antibody.The slides were then incubated with enzyme-conjugated secondary antibody at 37°C for 1 hour. Immunoreactivity was visualized using an enhanced diaminobenzidine (DAB) substrate kit (TransGen Biotech, Beijing, China), and nuclei were counterstained with hematoxylin.

### Cell lines and culture

The cell lines JHH-7, SNU-475, LO2, and THP-1 were obtained from the American Type Culture Collection. JHH-7 and SNU-475 cells were cultured in DMEM/F12 medium supplemented with 10% fetal bovine serum (FBS) and 1% penicillin/streptomycin (Invitrogen, USA). THP-1 cells were maintained in RPMI 1640 medium (Cytiva, USA) with the addition of 10% FBS. All cells were incubated in a humidified atmosphere at 37°C with 5% CO₂. Subculturing was performed at a ratio of 1:2 to 1:4, and the culture medium was replaced every 2–3 days.

### Cell transfection

After culturing the cells to 40% confluence in 6-well plates, they were transduced using a lentiviral vector (Genechem). After 24 hours, 3 mg/mL of puromycin was added for selection for 24 hours.

### RNA extraction and qRT-PCR

Total RNA was extracted from transfected cells using TRIzol reagent (Thermo Fisher Scientific), following the manufacturer's instructions.The relative expression of the STX7 gene was quantified by qRT-PCR, normalized to 18S rRNA, using the IQ5 multiplex real-time PCR system (Bio-Rad Laboratories, CA, USA).The PCR protocol consisted of 3 minutes of initial denaturation at 95°C, followed by 40 cycles of 15 seconds at 95°C, 15 seconds at 56.5°C, and 30 seconds at 72°C.The human STX7 primer sequences were: (Forward) 5’-GGCCCAGAGGATCTCTTCTAA’, (Reverse) 5’-ACTGTTGCCTCAATTCAGGTG-3’.The primer sequences for human 18S were: (Forward) 5’-AAACGGCTACCACATCCA-3’, (Reverse) 5’-CACCACTTGCCCCTCCA-3’.Relative expression levels were calculated using the 2^-ΔΔCT method.

### Western blotting

Protein extraction from cells was performed using RIPA buffer (Keygen, Nanjing, China) containing protease and phosphatase inhibitors. Protein concentration was determined using the BCA method, and samples were separated by SDS-PAGE and transferred to a nitrocellulose membrane. After transfer, protein bands were incubated with primary antibodies and visualized using enhanced chemiluminescence. GAPDH was used as a loading control. Throughout the study, the following primary antibodies were used: rabbit anti-human STX7 (ab224223, Abcam, UK), rabbit anti-human NF-κB p65 (#8242, Cell Signaling Technology), mouse anti-human p-NF-κB p65 (sc-136548, Santa Cruz Biotechnology), rabbit anti-human E-cadherin (#3195, Cell Signaling Technology), rabbit anti-human N-cadherin (#WL01047, Wanlei Biotechnology), rabbit anti-human MMP2 (#10373–2-AP, Proteintech), rabbit anti-human MMP9 (#10375–2-AP, Proteintech), and rabbit anti-human GAPDH (ab181602, Abcam, UK).

### Cell proliferation assay

The CCK-8 assay was used to evaluate cell proliferation. 6000 cells were seeded in a 96-well plate and cultured for 1, 2, or 3 days. Fresh medium (100 µL) was replaced before each experiment, followed by the addition of 10 µL CCK-8 reagent (Sigma). After 2 hours of incubation, the optical density (OD) at 450 nm was measured. In the EdU assay, 10 µM EdU was added to the medium, and cells were fixed and permeabilized after 2 hours of incubation. Following the manufacturer's instructions (ThermoFisher Scientific) for the Click-iT reaction, nuclear staining was performed using DAPI. Images were acquired using a fluorescence microscope (ThermoFisher Scientific). In the colony formation assay, 600 cells were seeded in a 6-well plate and cultured for 10 days. The cells were washed with PBS and fixed with 4% paraformaldehyde for 30 minutes.The cells were stained with 0.5% crystal violet at room temperature for 1 hour, and colonies were counted using a colony counting program.

### Wound healing assay

Hepatocellular carcinoma cells (1×10⁶) were digested with trypsin and seeded into each well of a 6-well plate. After overnight culture, a sterile pipette tip was used to create a scratch. The initial scratch width was captured, and the remaining width was measured 24 hours later.

### Macrophage infiltration assay

THP-1 cells were stimulated to differentiate into macrophages by treating them with 150 nM phorbol 12-myristate 13-acetate (PMA; Sigma, USA) for 24 hours. To perform macrophage infiltration assays, 1.0 × 10^5^ macrophages (200 μl) without serum were placed in the upper chamber of a Transwell plate and incubated for 48 hours. In the lower chamber, JHH-7 and SNU-475 cells (1.0 × 10^5^) were cultured in DMEM (800 μl) containing 10% FBS. After a 48-hour incubation, cells in the upper chamber were fixed with 4% paraformaldehyde and stained with 0.1% crystal violet. The number of infiltrating macrophages was determined by counting cells in three randomly selected fields under the microscope.

### In vivo tumor growth experiment

All animal experiments were approved by the Ethics Committee of Guangxi Zhuang Autonomous Region People's Hospital (Approval No. KY-GZR-2024-093) and conducted in accordance with their guidelines, including compliance with the ARRIVE guidelines. Mice were anesthetized with isoflurane and euthanized by CO₂ inhalation to minimize suffering and ensure humane endpoints. Five-week-old BALB/c nude mice (purchased from Beijing HuaFukang Biotechnology Co., Ltd., China) were used for the experiments. After random grouping, nude mice were subcutaneously injected with 200 μL of Matrigel suspension containing 1×10⁶ lentivirus-transduced JHH-7 cells (n = 5 per group).Tumor growth was monitored daily. After five weeks, the mice were euthanized, and the tumors were excised and measured.
