# Supplementary material for: Syntaxin-7 promotes EMT and tumor progression via NF-κB signaling and is associated with macrophage infiltration: pan-cancer analysis and experimental validation in hepatocellular carcinoma
Source: BMC Cancer. 2025 Sep 25;25:1430. doi: 10.1186/s12885-025-14819-0 (PMC12465986; doi:10.1186/s12885-025-14819-0)

Full-length Western blots showing STX7 expression in paired HCC tumor and adjacent non-cancerous tissues from five patients (Patient 1 − 5, from left to right).

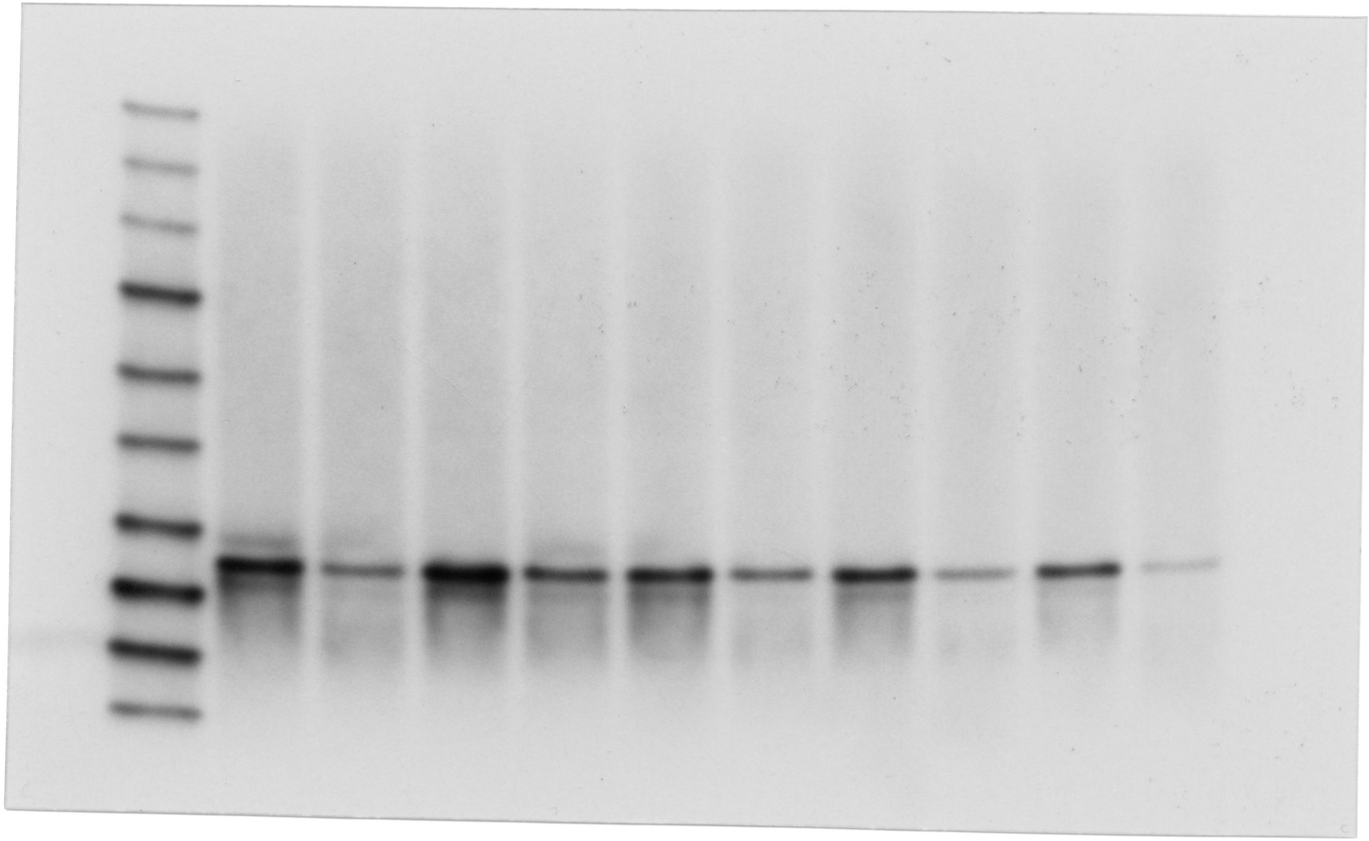

Full-length Western blots showing GAPDH expression in paired HCC tumor and adjacent non-cancerous tissues from five patients (Patient 1 − 5, from left to right).

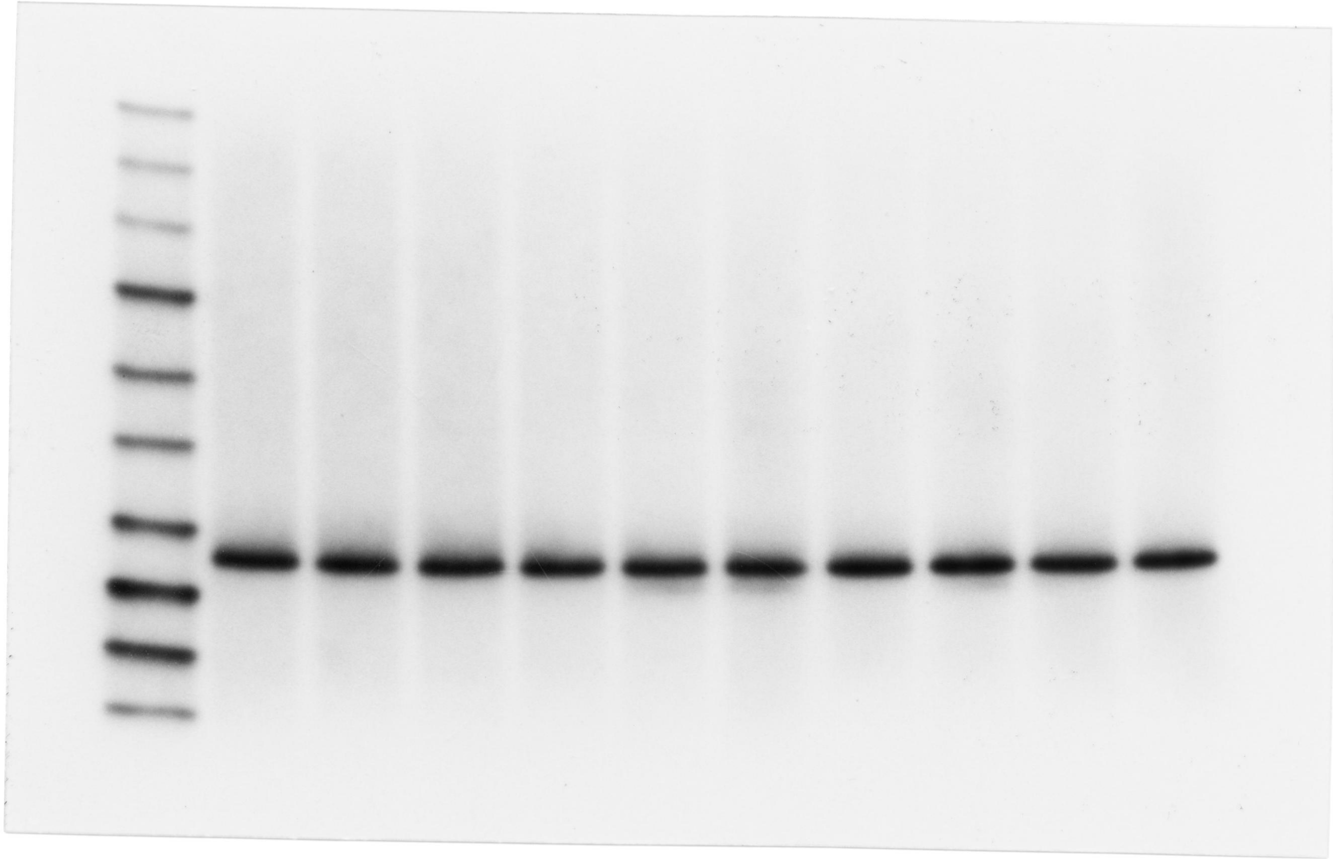

Full-length blots showing STX7 expression in liver cell line LO2, and HCC cell lines Hep3B, HepG2, HuH7, JHH-7, SUN398, SUN475 (from left to right).

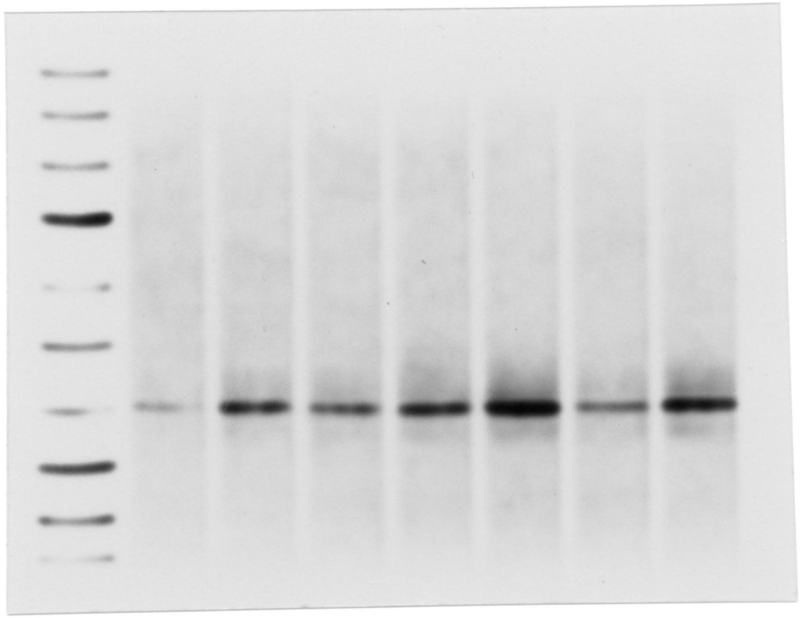

Full-length blots showing GAPDH expression in liver cell line LO2, and HCC cell lines Hep3B, HepG2, HuH7, JHH-7, SUN398, SUN475 (from left to right).

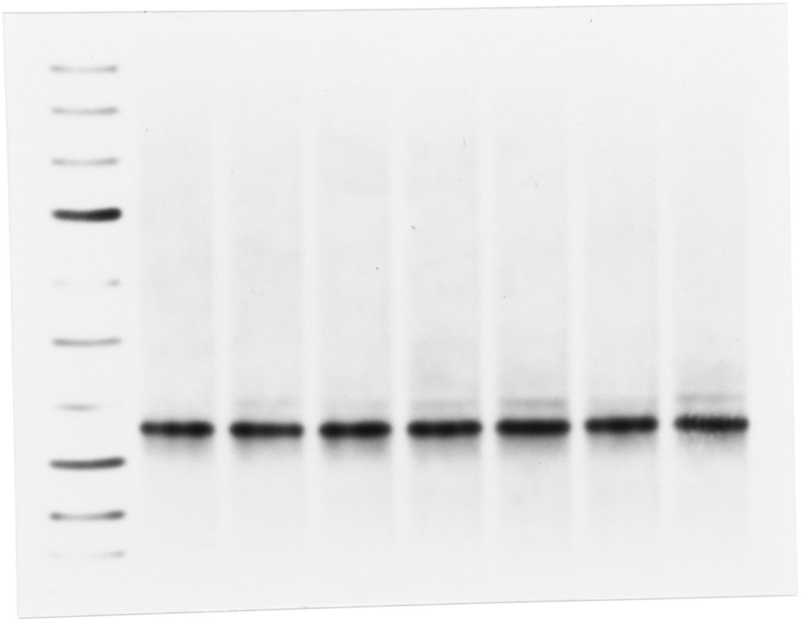

Full-length blots showing STX7 expression in SUN475 and JHH-7 cells following stable STX7 knockdown (from left to right: sh-NC SUN475, sh-STX7 SUN475, sh-NC JHH-7, sh-STX7 JHH-7).

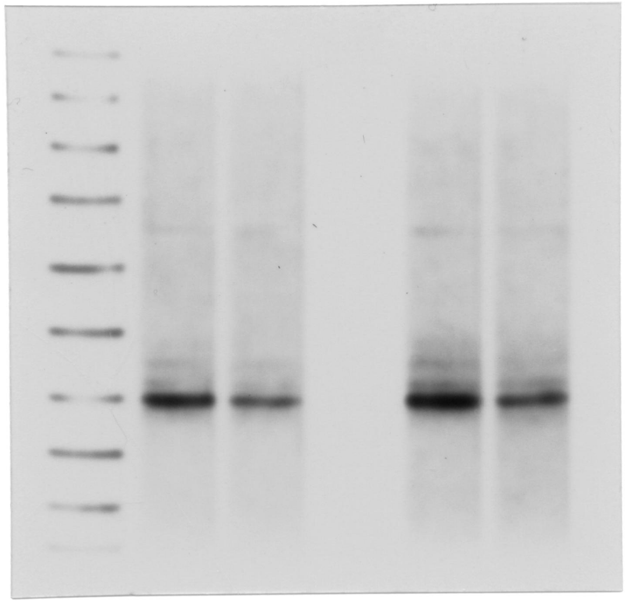

Full-length blots showing GAPDH expression in SUN475 and JHH-7 cells following stable STX7 knockdown (from left to right: sh-NC SUN475, sh-STX7 SUN475, sh-NC JHH-7, sh-STX7 JHH-7).

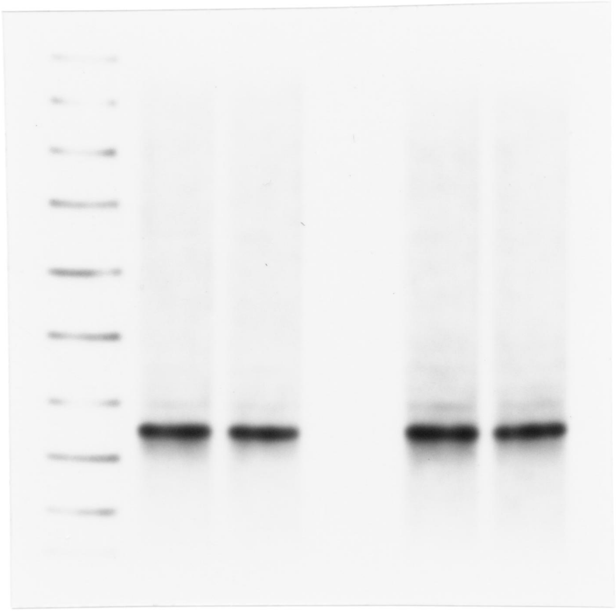

Full-length blots showing MMP9 expression in SUN475 and JHH-7 cells following stable STX7 knockdown (from left to right: sh-NC SUN475, sh-STX7 SUN475, sh-NC JHH-7, sh-STX7 JHH-7).

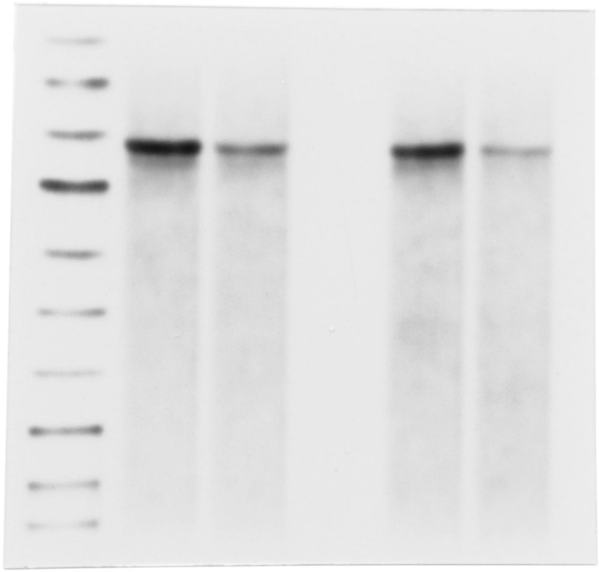

Full-length blots showing MMP2 expression in SUN475 and JHH-7 cells following stable STX7 knockdown (from left to right: sh-NC SUN475, sh-STX7 SUN475, sh-NC JHH-7, sh-STX7 JHH-7).

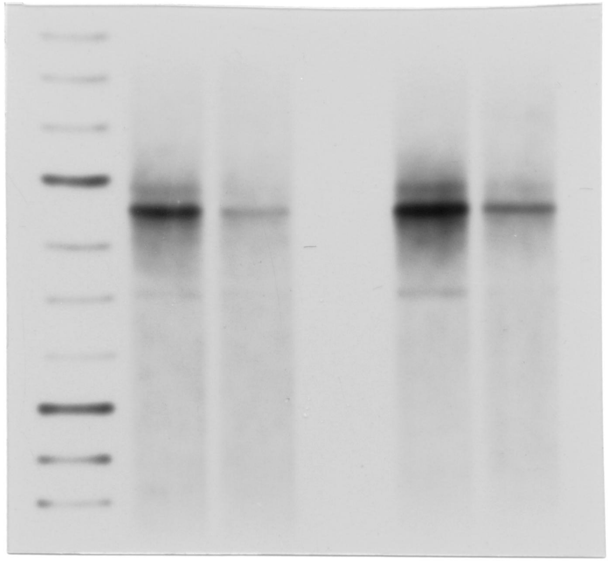

Full-length blots showing N-cadherin expression in SUN475 and JHH-7 cells following stable STX7 knockdown (from left to right: sh-NC SUN475, sh-STX7 SUN475, sh-NC JHH-7, sh-STX7 JHH-7).

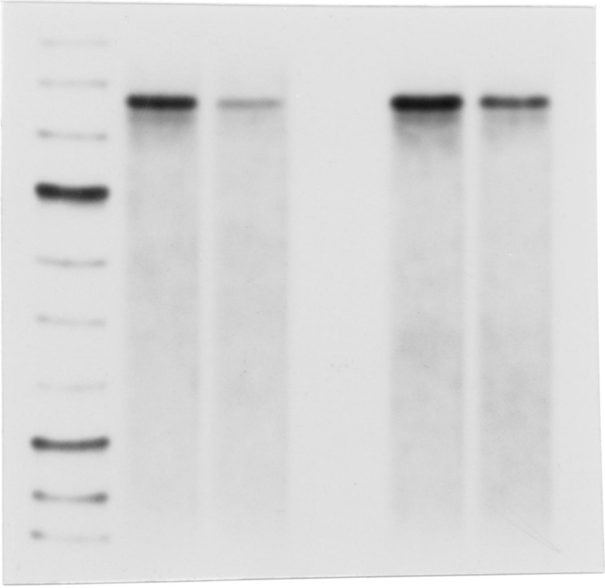

Full-length blots showing E-cadherin expression in SUN475 and JHH-7 cells following stable STX7 knockdown (from left to right: sh-NC SUN475, sh-STX7 SUN475, sh-NC JHH-7, sh-STX7 JHH-7).

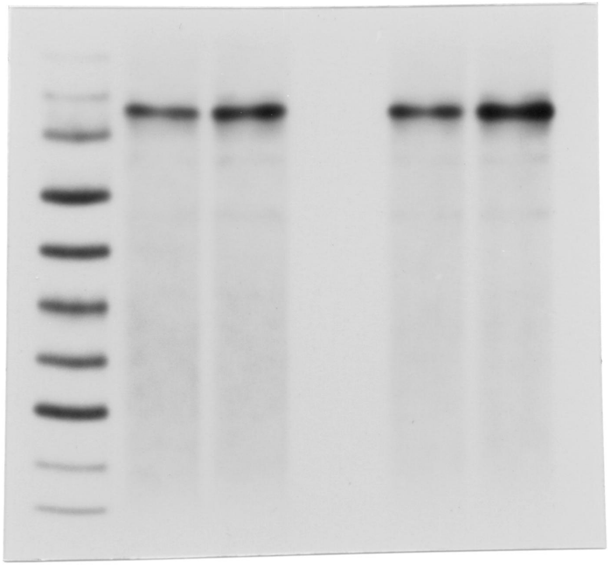

Full-length blots showing p-NF-κB P65 expression in SUN475 and JHH-7 cells following stable STX7 knockdown (from left to right: sh-NC SUN475, sh-STX7 SUN475, sh-NC JHH-7, sh-STX7 JHH-7).

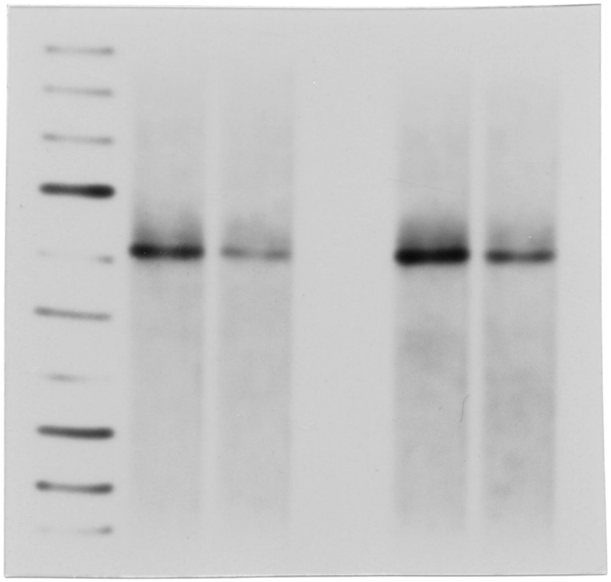

Full-length blots showing NF-κB P65 expression in SUN475 and JHH-7 cells following stable STX7 knockdown (from left to right: sh-NC SUN475, sh-STX7 SUN475, sh-NC JHH-7, sh-STX7 JHH-7).

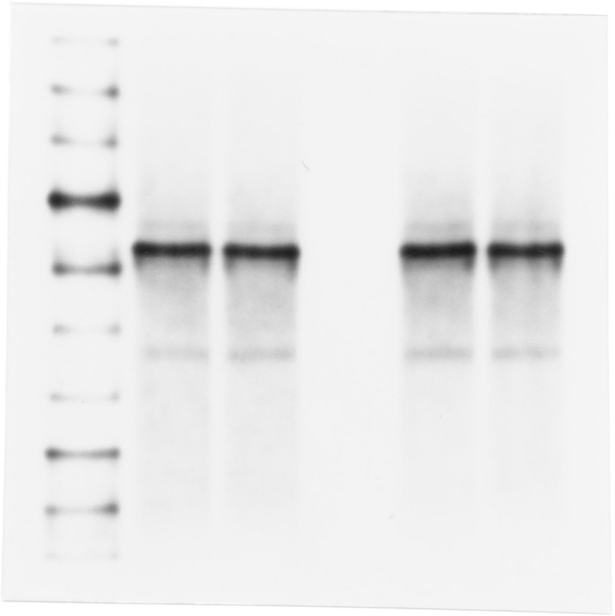

Full-length blots showing STX7 expression in SUN475 and JHH-7 cells following stable STX7 knockdown (from left to right: sh-NC SUN475, sh-STX7 SUN475, sh-NC JHH-7, sh-STX7 JHH-7).

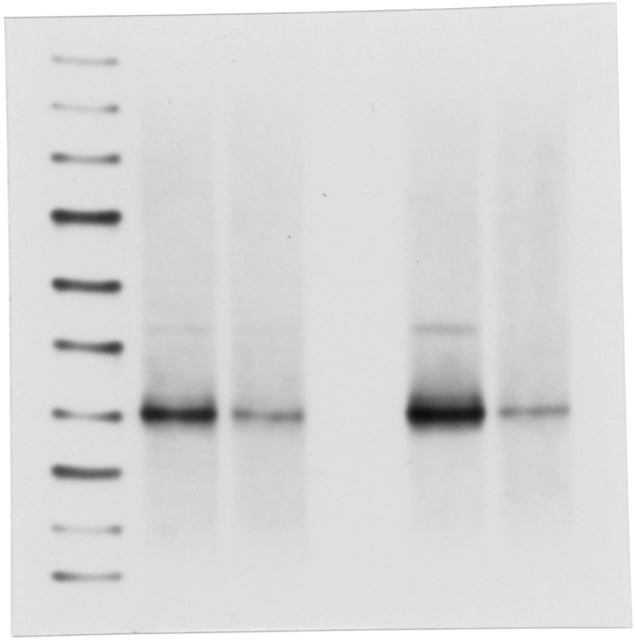

Full-length blots showing GAPDH expression in SUN475 and JHH-7 cells following stable STX7 knockdown (from left to right: sh-NC SUN475, sh-STX7 SUN475, sh-NC JHH-7, sh-STX7 JHH-7).

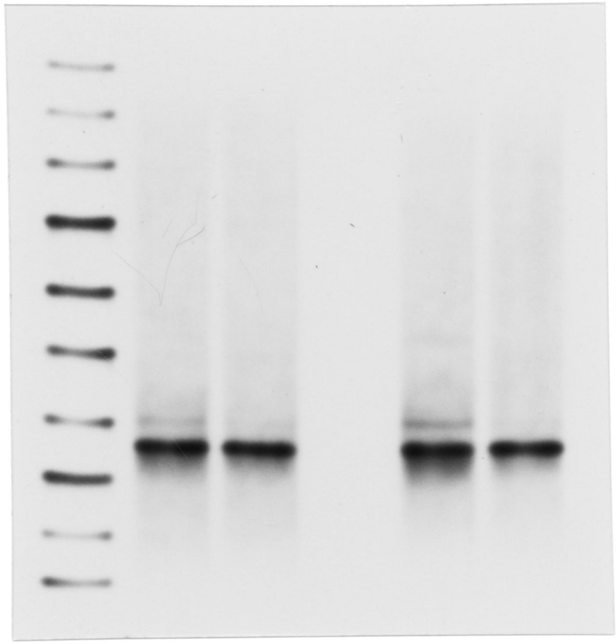

Supplement: Supplementary file 8 — Supplementary Material 8. [file 12885_2025_14819_MOESM8_ESM.pdf]
